# A Message from Thwaites

**Published:** 1923-03

**Authors:** 


### A MESSAGE FROM THWAITES

In our previous letters we took up the matter of safety of our machine, as well as its simplicity and ease of operation and certainty of results. It is, however, of the utmost importance that you have a unit of large capacity in order to get these results.

There is no other manufacturer of dental X-ray equipment that makes and uses a transformer as large as ours. This transformer has a capacity of 7″ back-up. It is made on an entirely different principle. It needs no grounding, which is an added safety feature. There are over forty miles of wire wound on each transformer. No other manufacturer could wind so large an amount of wire in so small a space, as the method of winding is patented by our company. We are enclosing a small sample of the wire we use. This may interest you as well as some of your prospective purchasers.

Our transformer is so constructed that it is not as sensitive to the fluctuation of line voltage as other transformers.

When using a Thwaites, it is just like driving a Pierce-Arrow, Packard or Cadillac in comparison with one of Henry's standbys, you always have the reserve power and never need to throw her into low.

The tube used is of the Coolidge Radiator Type and of the largest size manufactured, being a 5″ 30 M. A. tube. There is only one other X-ray manufacturer, and that is the manufacturer of the tube, who uses this size and type of tube in their dental X-ray units. This in itself should prove that it is the best type tube adapted for dental work. Other manufacturers can not use a tube of this size for dental work, as their transformers are not large enough to take care of same.

If it were not for our patented, simplified method of construction, we would not be able to build a unit, even at the new price, of such large capacity and of so high a grade, at anywhere near the price we are selling it for. We have eliminated many little features which only add to the cost and cause trouble both for the dealer and dentist, and which do not in any way add to the practicability of the unit, but rather detract from it, due to frequently getting out of order.

If you will just compare the features in the Thwaites (many of which it is impossible to obtain in any unit but a Thwaites) with those to be had in any other X-ray machine, you will find that ours is by far the best, regardless of price.

Do not forget we have an office here in Grand Rapids that is always open to suggestions and ready to back you up and help you in every way we can.

